# Multi-focal Lytic Lesions in a Patient with Myelofibrosis: A Case Report

**DOI:** 10.7759/cureus.7475

**Published:** 2020-03-30

**Authors:** Robert R Burnham, Bailey Johnson, Laurie M Lomasney, Dariusz Borys, Anna R Cooper

**Affiliations:** 1 Orthopaedic Surgery, Loyola University Medical Center, Maywood, USA; 2 Musculoskeletal Radiology, Loyola University Medical Center, Maywood, USA; 3 Pathology, Loyola University Medical Center, Maywood, USA; 4 Orthopaedic Surgery & Rehabilitation, Loyola University Medical Center, Maywood, USA

**Keywords:** myelofibrosis, osteolytic lesion, bone lesion, metastatic, myeloproliferative disorder

## Abstract

Myelofibrosis is a rare disorder that is classified as one of the myeloproliferative disorders. This particular disorder results in the abnormal proliferation of hematopoietic stem cells in the bone marrow. In some cases, such as ours, pathologic fractures can occur due to skeletal manifestations. We report on a rare finding of rapidly progressive lytic lesions in multiple regions throughout the body. This presentation of myelofibrosis behaving in a metastatic-like fashion has not been previously described.

## Introduction

Myelofibrosis is a myeloproliferative disorder characterized by the abnormal proliferation of hematopoietic stem cells within the bone marrow, which leads to overproduction of fibrous tissue. Uncommonly, extramedullary hematopoiesis can occur, which can lead to systemic effects on bone, internal organs, and the vascular system. The disease manifestations and progression are variable, which could include splenomegaly, bone pain, leukocytosis, thrombocytosis, infections, conversion to acute myeloid leukemia, and/or constitutional symptoms [1]. The incidence of myelofibrosis is approximately 0.1 to 1 per 100,000 individuals per year with the median age of presentation at 64 years old [2]. The survival rate following a diagnosis of myelofibrosis is variable and many scoring systems exist in an attempt to quantify the average years of survival following diagnosis. These scoring systems provide a risk group score based on some of the following factors: patient’s age, hemoglobin level, white blood cell count, presence of constitutional symptoms, platelet level, genetic mutations, and percentage of myeloblasts within the peripheral blood [3]. The presence of lytic bone lesions in these patients has only been described in a handful of case reports throughout the literature.

We report on a case of myelofibrosis that behaved in a rare, malignant-like spread of lytic bone lesions throughout the body within a one-year time frame. In addition, we describe other cases throughout the literature that share some similarities to our own case presentation.

## Case presentation

**Figure 1:**
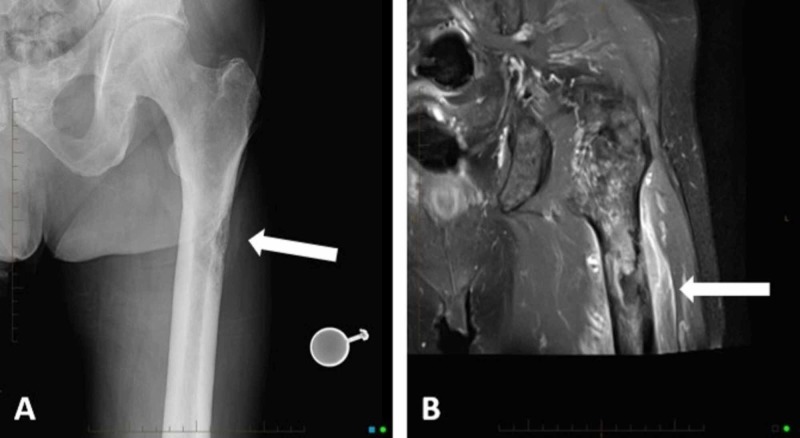
Left femur imaging. A plain radiograph of the left proximal femur identifies a sub-trochanteric permeative, lytic bone lesion with cortical destruction (A), which is re-demonstrated on a T2-weighted fat-saturated MRI of the left proximal femur with notable intramedullary fibrosis, marked heterogeneity of the marrow, and associated soft tissue edema surrounding the proximal femur (B).

A 63-year-old male initially presented for orthopedic evaluation after experiencing one month of left lateral hip and thigh pain. His past medical history includes JAK2+ myelofibrosis, which was diagnosed three years prior and treated with splenectomy. He also underwent a bone marrow transplant one year before his current presentation and was placed on chronic immunosuppression. Notably, this patient was very physically active and ran a five-kilometer race one month prior to his presentation. On physical exam, his pain was reproducible when bearing weight on his left lower extremity. He was found to be anemic with a hemoglobin of 8.9 g/dL (two months prior 13.0 g/dL) and also in a state of thrombocytosis with a platelet count of 544 K/uL. Full length femur radiographs were obtained and demonstrated a permeative, lytic lesion of the proximal femur that involved the medullary canal and lateral cortex (Figure 1A). Advanced imaging provided further evidence of the lesion’s involvement into the surrounding soft tissue (Figure 1B).

**Figure 2:**
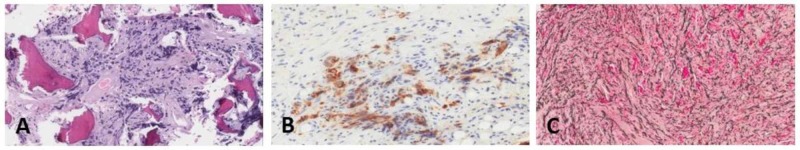
Left femur histologic sections. Histologic section (A), immunohistochemistry stain for CD61 (B), and reticulin stain (C) of the left proximal femur.

An open biopsy of the femoral lesion was performed. Histologic sections of the biopsy tissue showed bone marrow fibrosis with increased megakaryocytes with some displaying hyperchromatic nuclei. Bone trabeculae showed osteosclerotic change and remodeling (Figure 2A). Immunohistochemistry stain for CD61 highlights megakaryocytes (Figure 2B) and reticulin stain demonstrates extensive marrow fibrosis (Figure 2C). Thus, the final diagnosis of myelofibrosis was concluded.

The patient underwent left femur intramedullary nail fixation followed by palliative radiation therapy, 25 Gy in 10 fractions. Two months after fixation of the left femur, he experienced new right hip and thigh pain. This new pain was not reproducible with weight-bearing. Full length right femur radiographs were obtained that showed lateral cortical thickening of the proximal femur with mottled bone demineralization. Advanced imaging showed identical findings to his myelofibrotic lesion on the contralateral side. The decision was made to not prophylactically fix his right femur because there was no cortical destruction seen on imaging and he did not have any mechanical pain. This lesion was managed with radiation therapy, 25 Gy in 10 fractions.

**Figure 3:**
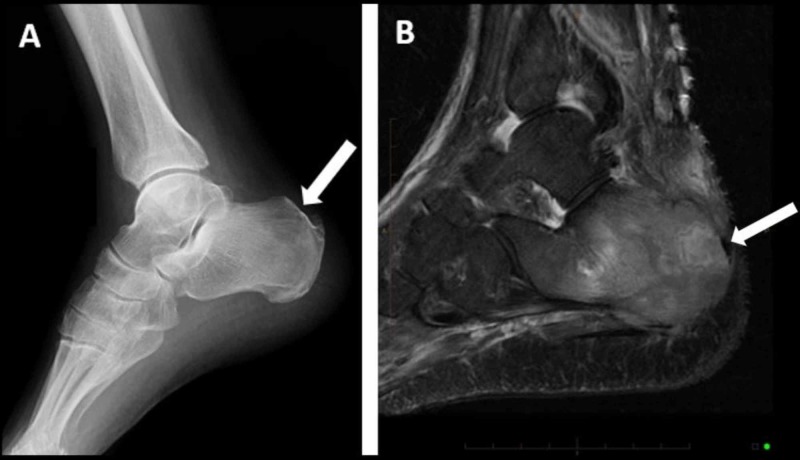
Right calcaneus imaging. Plain lateral radiograph of the right ankle with a posterior superior calcaneal lucency approximately 2 cm in diameter (A) and a T2 fat saturation weighted sagittal MRI of the right calcaneal lesion further demonstrating a destructive, lytic lesion (B).

During radiation treatment for his right proximal femur, he developed progressively worsening right heel pain. Over several months, it became swollen and allodynic. Radiographs demonstrated a subtle, postero-superior calcaneal lucency (Figure 3A). Advanced imaging provided further demonstration of a permeative lesion on the right, posterior calcaneus with cortical destruction and a medial soft-tissue mass (Figure 3B). An ultrasound-guided biopsy was performed, which confirmed a myelofibrotic lesion. He completed radiation therapy, 20 Gy in 10 fractions, with symptomatic improvement.

**Figure 4:**
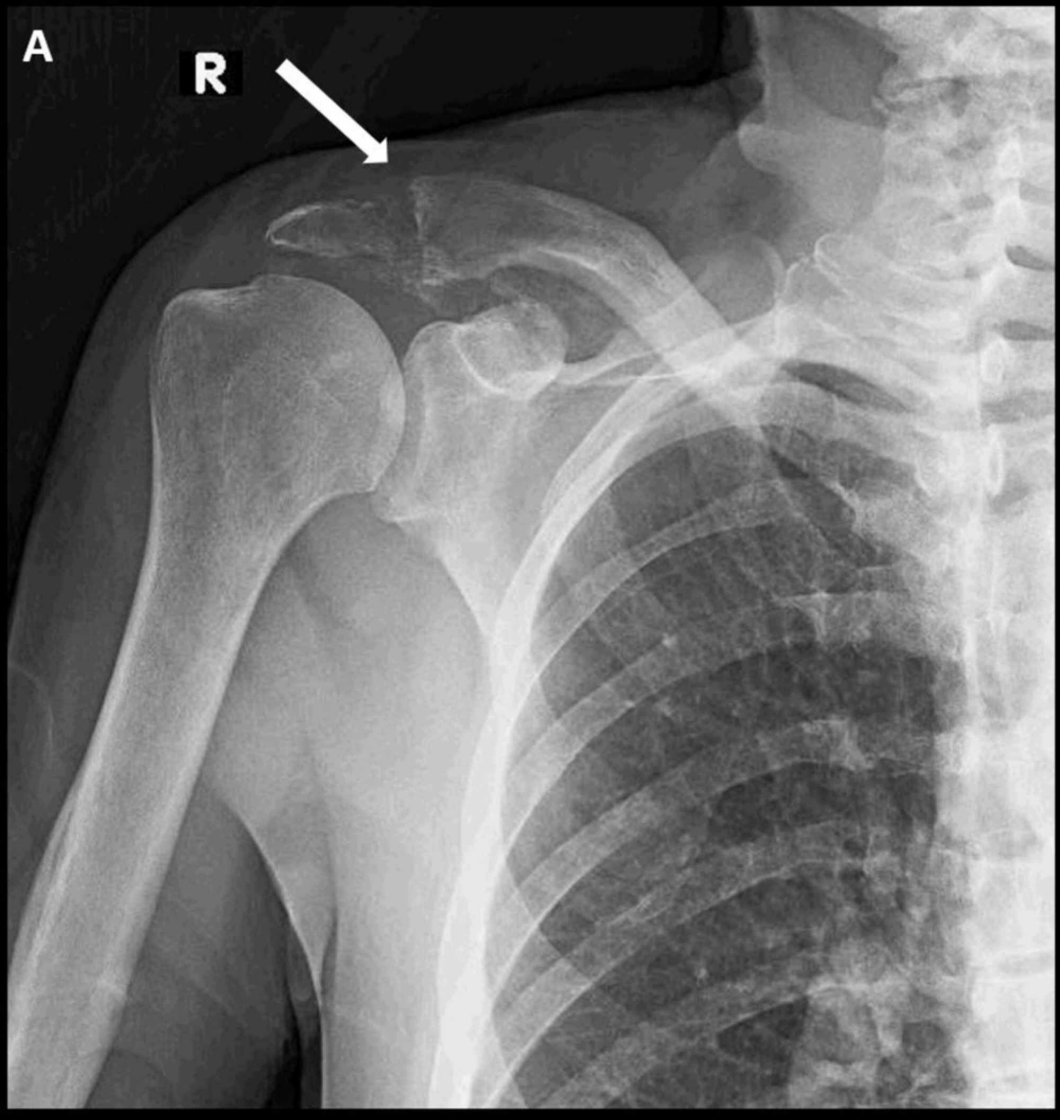
Right shoulder radiograph. A plain radiograph of the right shoulder identifying a permeative, lytic lesion of the distal clavicle and acromion (A).

Eight months from his original presentation, he developed right shoulder pain. Radiographs were obtained and an acromio-clavicular lesion was noted (Figure 4). Due to the progressive multi-focal bony lesions and short interval between presentations, ruxolitinib was initiated for systemic treatment. His shoulder pain improved with one month of physical therapy and medical management. 

**Figure 5:**
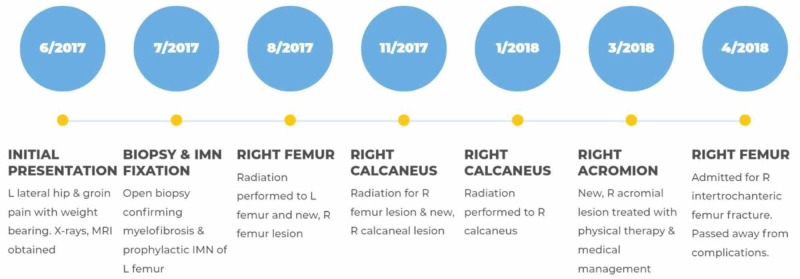
Case timeline. This timeline summarizes a fairly aggressive time frame starting from initial presentation to the patient passing away from complications related to his myelofibrosis.

Two months later he was admitted to the hospital after a low energy fall and sustained a right intertrochanteric femur fracture. Prior to fixation, the patient began to clinically decline and was intubated secondary to severe acidosis. He continued to decline, requiring dialysis for kidney failure and vasopressors for hypotension. The decision was made to withdraw care and transition to palliative care. Just 10 months after his initial presentation, the patient passed away. A timeline of the patient's clinical course is displayed in Figure 5.

## Discussion

Myelofibrosis is the least common chronic myeloproliferative disorder and primarily impacts adults over age 50 [4]. Extra-osseous manifestations include hepatomegaly and/or splenomegaly, fatigue, B symptoms, and less commonly thrombotic events, pulmonary hypertension, and conversion to acute leukemia. Laboratory abnormalities may include anemia, grossly elevated circulating CD34+ hematopoietic precursor cells (can be found in levels 400x that of normal), elevated alkaline phosphatase, lactate, B12, and uric acid levels [4-10]. Genetic mutations are found in nearly 90% of patients, most commonly JAK2 (as with our patient), though abnormalities can also be found in calreticulin (CALR) and myeloproliferative leukemia (MPL) genes [11].

Bone and joint symptoms include gout, periostitis, and generalized bone pain due to hypermetabolism and elevated circulating uric acid levels caused by high hematopoietic tissue turnover. Fibrotic tissue replaces the 30%-70% cellular composition of normal marrow resulting in extramedullary hematopoiesis [12]. Osteosclerosis is common (found in 25%-66% of patients) and the increased trabecular prominence and mottled appearance on plain films can be confused for metastatic carcinoma [13].

Lytic lesions are a rare complication of myelofibrosis. Lytic lesions are typically found to be solid collections of leukemic cells, i.e. solitary myeloid sarcoma or chloroma [14]. Though common in multiple myeloma and malignancy, only four other published cases of pathologically benign lytic lesions in patients with myelofibrosis were found (in English speaking literature). In one case a 59-year-old male had a stable myelofibrosis of 33 years before a lytic lesion of his femoral shaft appeared. The femur was prophylactically stabilized, but the patient passed within one year secondary to complications associated with pneumonia. The other three case reports were of patients in their 60s or older who initially presented with fatigue, hepatomegaly, and splenomegaly. They had osteolytic lesions, but the progression of their disease was over the course of 10 or more years before passing away due to complications from their myelofibrosis [15-18].

## Conclusions

The presence of osteolytic lesions from myelofibrosis is a finding that is rarely encountered. More commonly, osteoblastic or osteosclerotic type lesions are seen due to the ossification of the fibrotic processes that occur within the bony trabeculae. When osteolytic lesions are seen, it is important for the clinician to search for other possible etiologies such as carcinoma, multiple myeloma, or lymphoma. Our patient developed osteolytic lesions in four different areas of the body and passed away from complications all within one year of his initial presentation. It appears that the presence of osteolytic lesions leads to a worse prognostic outcome. Our specific case carried a poor prognosis like the others, but was unique in its multi-focal presentation and metastatic-like behavior within a 10-month time frame.
